# Lignocellulose degradation in isopods: new insights into the adaptation to terrestrial life

**DOI:** 10.1186/s12864-019-5825-8

**Published:** 2019-06-07

**Authors:** Marius Bredon, Benjamin Herran, Baptiste Lheraud, Joanne Bertaux, Pierre Grève, Bouziane Moumen, Didier Bouchon

**Affiliations:** Laboratoire Ecologie et Biologie des Interactions - UMR CNRS 7267, Equipe Ecologie Evolution Symbiose - Bâtiment B8-B35, Université de Poitiers, 5 rue Albert Turpain, TSA 51106, F-86073 Poitiers Cedex 9, France

**Keywords:** Transcriptomics, CAZymes, Isopods, Lignocellulose, Terrestrialization

## Abstract

**Background:**

Isopods constitute a particular group of crustaceans that has successfully colonized all environments including marine, freshwater and terrestrial habitats. Their ability to use various food sources, especially plant biomass, might be one of the reasons of their successful spread. All isopods, which feed on plants and their by-products, must be capable of lignocellulose degradation. This complex composite is the main component of plants and is therefore an important nutrient source for many living organisms. Its degradation requires a large repertoire of highly specialized Carbohydrate-Active enZymes (called CAZymes) which are produced by the organism itself and in some cases, by its associated microbiota. The acquisition of highly diversified CAZymes could have helped isopods to adapt to their diet and to their environment, especially during land colonization.

**Results:**

To test this hypothesis, isopod host CAZomes (i.e. the entire CAZyme repertoire) were characterized in marine, freshwater and terrestrial species through a transcriptomic approach. Many CAZymes were identified in 64 isopod transcriptomes, comprising 27 de novo datasets. Our results show that marine, freshwater and terrestrial isopods exhibit different CAZomes, illustrating different strategies for lignocellulose degradation. The analysis of variations of the size of CAZy families shows these are expanded in terrestrial isopods while they are contracted in aquatic isopods; this pattern is probably resulting from the evolution of the host CAZomes during the terrestrial adaptation of isopods. We show that CAZyme gene duplications and horizontal transfers can be involved in adaptive divergence between isopod CAZomes.

**Conclusions:**

Our characterization of the CAZomes in 64 isopods species provides new insights into the evolutionary processes that enabled isopods to conquer various environments, especially terrestrial ones.

**Electronic supplementary material:**

The online version of this article (10.1186/s12864-019-5825-8) contains supplementary material, which is available to authorized users.

## Background

Plant biomass is the most abundant source of renewable carbon on earth [1]. Mostly composed of lignocellulose, it is an important resource for many organisms. Its decomposition involves the combined action of fungi, microbes and decomposer animals such as “litter transformer” macroarthropods [2, 3]. Among them, terrestrial isopods (Oniscidea) are known to contribute directly to litter decomposition and nutrient cycling by digesting substrates [4–9], and indirectly through their faeces which affect the soil microbial community and its activity [10–12]. From marine ancestors, terrestrial isopods have successfully colonized all environments including freshwater and terrestrial habitats [13]. It is assumed that their ability to use plant biomass as a food source facilitated their colonization of terrestrial environments in the Late Paleozoic (~ 300 Ma, Permo-Carboniferous), together with morphological and physiological adaptations [14–17]. Their digestive tract consists of a short foregut comprising an esophagus and a stomach, a hindgut and a hepatopancreas where endogenous digestive enzymes are secreted; it allows an efficient digestion of their food [18, 19]. Lignocellulose constitutes the main food not only of terrestrial isopods, but also of freshwater isopods (Aselotta) which feed on plant detritus of terrestrial origin [20, 21]. Many marine isopods (e.g. Valvifera, Sphaeromatidea and Limnoriidae) also consume cellulose and hemicellulose that arises, for example, from algae or driftwood [17].

Few studies have characterized enzymes which participate in lignocellulose decomposition in isopods. Kern et al. [22] have characterized an exoglucanase in the marine isopod *Limnoria quadripunctata* and Kostanjnek et al. [23] have highlighted the endogenous production of an endoglucanase in the terrestrial isopod *Porcellio scaber*. These enzymes, identified as Carbohydrate Active enZymes (CAZymes) and classified as GH7 and GH9 in the families of the CAZy database [24], belong to a complex pathway involving many CAZymes that can degrade and release monosaccharides from lignocellulose. Recently, Bredon et al. [25] have identified 17 endogenous lignocellulose-degrading CAZy families in the common pill-bug *Armadillidium vulgare,* which illustrates the complexity of this process in this terrestrial isopod. Furthermore, some marine isopods could use hemocyanins in their digestive tract to modify lignin during lignocellulose digestion, thus facilitating access to cellulose and hemicellulose [26, 27].

However, there is so far no animal genome known to encode all necessary CAZymes to degrade lignocellulose, and in most cases, animals benefit from mutualistic associations with microbial symbionts allowing an efficient degradation of lignocellulose [28]. In arthropods like termites [29, 30] and beetles [31, 32], there is a complementary and synergistic action of the lignocellulose-degrading enzyme repertoire from the host and its associated microbial symbionts. The host and its microbiota achieve the degradation of the three lignocellulose components (i.e. cellulose, hemicellulose and lignin) in a cooperative manner in different parts of the digestive system. This is also the case for terrestrial isopods (Oniscidea), which interact with their microbiota to digest lignocellulose [16, 33–37]. This microbiota is mostly composed of hepatopancreas-resident bacteria and environmental bacteria localized in the hindgut [25]. It completes the set of lignocellulose-degrading enzymes that the host produces mostly in the hepatopancreas. Both marine and freshwater isopods belonging to the suborder Asellota share this ability with terrestrial isopods: they contain hepatopancreatic bacteria that contribute to the digestion of their food [20, 36]. However, several studies show that some marine isopods belonging to the suborders Valvifera, Sphaeromatidea and Limnoriidea, digest cellulose without the help of hepatopancreatic and gut bacteria [22, 36–39]. Strategies for lignocellulose digestion may therefore differ between isopods species according to their biotopes and interactions between hosts and their microbiota. Moreover, the ability to digest lignocellulose varies in isopods, the terrestrial species being the most efficient ones [20]. Thus, the host CAZome (i.e. the CAZyme repertoire encoded in the host’s genome) is expected to be different across isopods, according to their evolutionary history.

In the present study, we identified and annotated the host CAZomes of many isopod species belonging to different families to better understand their successful colonization of all environments. To this end, we identified CAZymes from 64 isopod transcriptomes, including both publicly available transcriptomes and 27 de novo sequenced and assembled datasets. These transcriptomes were obtained from a wide range of isopod species among Oniscidea, Asellota, Valvifera, Sphaeromatidea and Limnoriidea. Our analysis of the CAZomes from this large dataset highlights the different strategies for lignocellulose digestion in isopods, according to their adaption to marine, freshwater and terrestrial environments. In addition, we assessed the molecular evolution of some lignocellulose-degrading key genes, suggesting an ancient origin and acquisition of these genes during the conquest of land.

## Results

### Transcriptome assemblies

In total, 64 transcriptomes of isopod species, including 27 new transcriptomes, were assembled. The resulting assemblies ranged from 28,393 (*Jaera hopeana*) to 685,588 (*Limnoria tripunctata*) transcripts depending on the species (Additional file 1). Assembly completeness was evaluated using the BUSCO pipeline. Except for some *Proasellus* species, all the transcriptomes displayed a good completeness since more than 80% of the complete genes from the arthropod core genome were present in most of the assemblies (Additional file 2).

### Distribution of the CAZy families

**Fig. 1:**
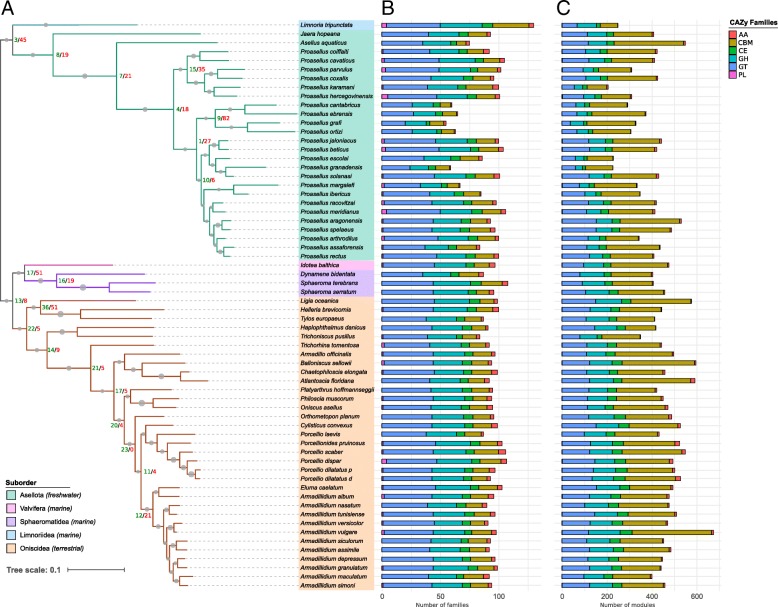
Dynamic evolution of isopod’s CAZomes. **a** Gene family expansion and contraction in each evolutionary branch. The species tree was constructed with the STAG method. The colours of the branches indicate the Isopoda suborders. Circles are proportional with the bipartition support values. The numbers of expected gains (green) and losses (red) of CAZymes are shown at the node of divergence. These numbers were calculated by CAFE considering the most likely CAZy family size at all internal nodes. **b** Number of CAZy families found in transcriptomes. **c** Normalized count of CAZy modules found in transcriptomes

**Fig. 2:**
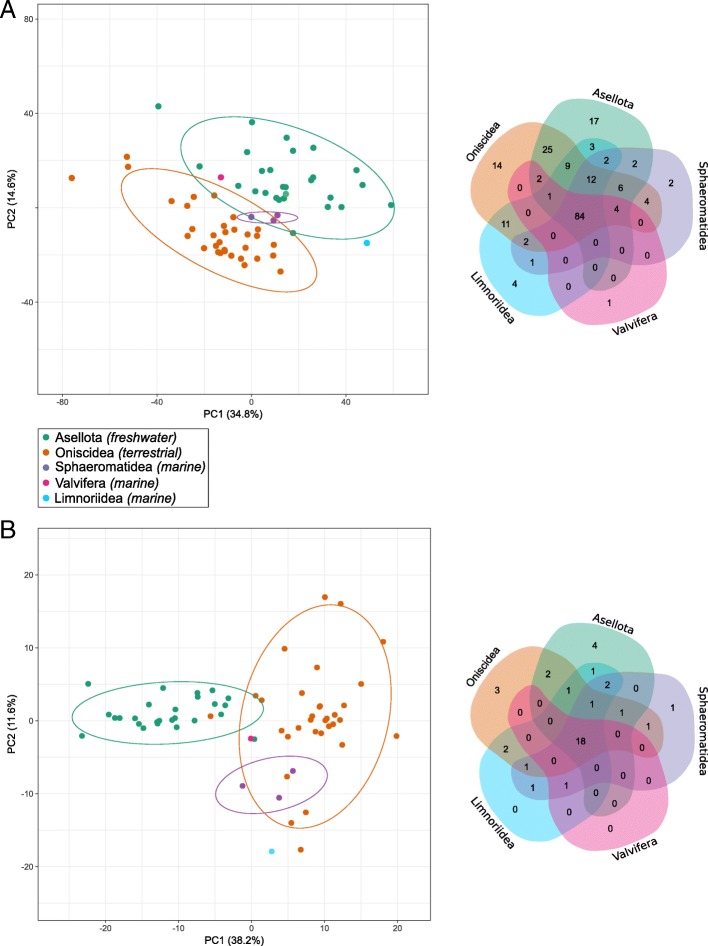
Comparative analysis of isopod CAZomes comprising all CAZymes (**a**) and lignocellulose degrading CAZymes only (**b**). The PCA was constructed from normalized counts of CAZy modules and the Venn diagram was constructed from numbers of CAZy families identified in the transcriptomes

In total, 205 CAZy families were identified with an average of 93 families *per* isopod species (Additional files 3 and 4). Carbohydrate-Binding Modules (CBMs), Carbohydrate Esterases (CEs), Glycoside Hydrolases (GHs) and Glycosyl Transferases (GTs) were present in all isopod transcriptomes (Fig. 1). The CBM modules were the most numerous (334 modules on average per transcriptome), followed by the GT, GH and CE modules (on average 184, 135 and 59 per transcriptome, respectively). Polysaccharide Lyases (PLs) were absent in one third of the transcriptomes whereas Auxiliary Activities (AAs) were absent from the transcriptomes of *Proasellus margalefi*, *Proasellus grafi*, *Proasellus ebrensis* and *Proasellus cantabricus* (Fig. 1). When they were present, they were in small numbers (on average 8 and 1 modules per transcriptome, respectively). Despite this feature which might be due to the low coverage in the transcriptomes involved, the distribution of the CAZymes appeared quite homogeneous across isopods. However, multivariate analysis (PCA) showed that CAZomes were different between isopod suborders (Fig. 2a) as only 84 CAZy families were shared by all isopod species. The transcriptomes of Asellota, Limnoriidea, Oniscidea, Sphaeromatidea and Valvifera contained a lot of specific CAZy families.

Asellota, Valvifera and Sphaeromatidea were dominated by CAZyme loss, whereas Oniscidea were dominated by CAZyme gains (Fig. 1, Additional file 4). The terrestrial isopods (Oniscidea) clade showed 22 expanded and 5 contracted CAZy families compared to the ancestral birth and death rate of CAZy families predicted for the node grouping Valvifera, Sphaeromatidea and Oniscidea. In comparison, the node grouping Valvifera and Sphaeromatidea marine isopods showed 17 expanded and 51 contracted families. Similarly, the node grouping the marine isopod *Limnoria tripunctata* and Asellota freshwater isopods had larger numbers of contracted CAZy families than expanded ones (45 and 3 respectively) compared to the common ancestor of the 5 suborders. These trends were continued since Oniscidea showed a high number of expanded families compared to contracted families at almost all internal nodes, while Asellota were dominated by CAZy families’ contractions. Interestingly, among the 31 rapidly evolving CAZy families predicted by CAFE (Family-wide *P*-value < 0.01; Additional file 4), seven are known to participate to the lignocellulose degradation, including hemicellulases (CE3, GH29, GH31, GH35, GH38), and some members of GH30s that are recognized as cellulases or hemicellulases, and cellobiose dehydrogenases (AA3).

### Lignocellulose degrading CAZymes in isopods

**Fig. 3:**
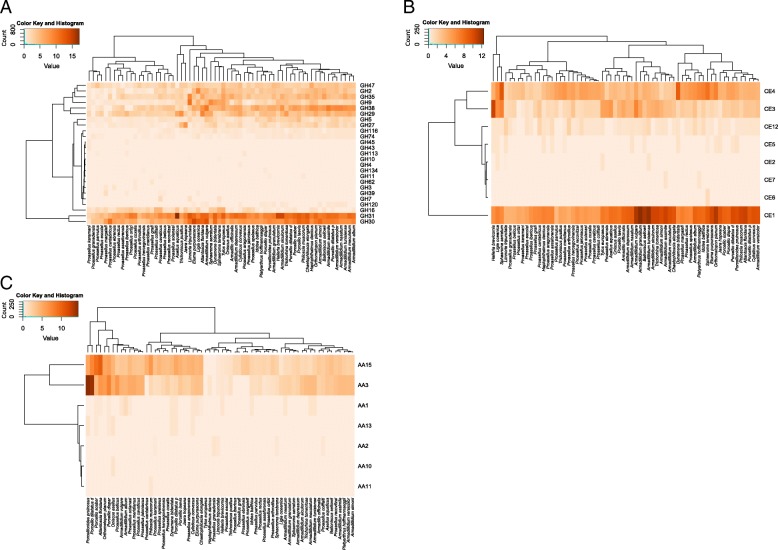
Abundances of lignocellulose-degrading CAZymes in the transcriptomes. Heatmaps of GH families (**a**), CE families (**b**) and AA families (**c**) were created from normalized counts of CAZy modules

Given that not all CAZymes participate in lignocellulose degradation, lignocellulose degrading CAZymes were then examined in depth. Forty CAZy families were identified as lignocellulose degrading CAZymes in our transcriptomes, comprising AAs, GHs and CEs (Fig. 3). Among them, 18 were found in all isopod suborders. Despite this apparent functional redundancy, CAZomes linked to lignocellulose degradation were different across isopod species (Fig. 2b). Some CAZymes, including cellulases (GH5, GH9, GH30) and hemicellulases (GH27, GH29, GH30, GH31, GH35, GH47, CE1, CE3 and CE4), were found in more than 95% of the transcriptomes. The families GH31, GH30 and CE1 were the most abundant ones (Fig. 3a-b, Additional file 4), with 1088, 905 and 823 modules respectively identified in the transcriptomes. Lignin modifying enzymes (LMEs) and oxidative cellulases belonging to AA families were rarer in isopods; the most abundant of these AA families was AA3 with 333 modules distributed in 57 species, followed by AA1 with 18 modules identified in 16 species (Fig. 3c, Additional file 4). Additionally, we identified 342 modules distributed among 61 species belonging to AA15, a CAZy family recently characterized in arthropods [40]. In many arthropods AA15s are likely involved in chitin modification, but some of them could have expanded this family for cellulose digestion [40].

**Fig. 4:**
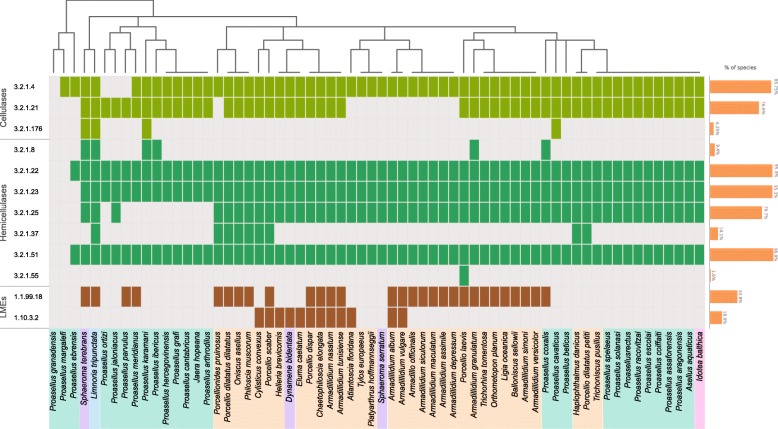
Prediction of enzymatic functions (EC number) of cellulases, hemicellulases and LMEs identified in the transcriptomes. Coloured squares indicate the presence of a given enzymatic function in a transcriptome. Horizontal bars show the proportion of all transcriptomes with the presence of each enzymatic function

The prediction of enzyme activities with Hotpep showed that cellulases and hemicellulases are well distributed in isopod species (Fig. 4). Cellulases were found in almost all transcriptomes, notably endo-β-1,4-glucanases (EC 3.2.1.4) that were predicted in more than 93% of the transcriptomes and β-glucosidases (EC 3.2.1.21) in 76% of the transcriptomes. Similarly, hemicellulases, mannases (EC 3.2.1.22) and xyloglucanases (EC 3.2.1.23 and EC 3.2.1.51) were predicted in more than 95% of the transcriptomes. In contrast, LMEs and oxidative cellulases seemed to be exclusive to Oniscidea and Sphaeromatidea. Cellobiose dehydrogenases (EC 1.1.99.18) and laccases (EC 1.10.3.2) were predicted in most of terrestrial and marine isopods, and were not found in freshwater isopods, except for *P. jaloniacus* and *P. parvulus*.

### GH9 and AA3 phylogenies

**Fig. 5:**
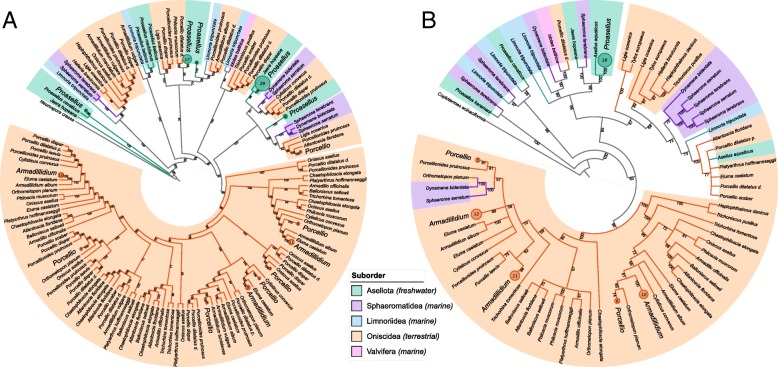
AA3 (**a**) and GH9 (**b**) gene phylogenies. The colours of the branches indicate the Isopoda suborder. Fast bootstrap values (*n* = 1000) for branches are shown as percentages. To facilitate reading, circles materialize collapsed branches and the numbers within refer to the number of collapsed branches. The corresponding species were abbreviated through their genera (*Proasellus sp.*; *Porcellio sp.*; *Armadillidium sp.*)

The GH9 enzymes are well studied in arthropods and many of them are known to participate in cellulose degradation. By contrast, AA3 enzymes are of interest because they are not widespread in animals and their role in lignocellulose degradation remains vague in arthropods. The phylogenies of AA3 and GH9 genes show that the CAZymes from different isopod suborders were grouped as distinct clusters (Fig. 5). In addition, the sequences belonging to a given species were spread across several branches of the tree as discrete sub-clusters, suggesting different origins. The alignments of nucleotide sequences from a given species showed a percent identity lower than 80% on average. This divergence is too small to correspond to alternative splicing events and suggests instead multiple gene duplications that occurred repeatedly within the isopod order.

The AA3 genes showed significant (BUSTED likelihood ratio test, *p*-value < 0.05) signatures of gene-wide episodic diversifying selection in the whole phylogeny (252 sequences) as well as in the subtree (131 sequences) comprising all the AA3 genes from terrestrial isopods (Additional file 5). Furthermore, all sequences showed evidence of positive selection at individual sites. SLAC found evidence of pervasive positive selection on one site (#1128, *p*-value = 0.088, Additional file 5) and negative selection on 630 sites (Additional file 5) whereas FUBAR found positive selection on 5 sites (#957, #1022, #1042,#1059 and #1076, posterior probability > 0.9, Additional file 5) and negative selection on 649 sites among 1277.

Unlike AA3 there is no evidence that any sites have experienced diversifying selection in the branches of the GH9 phylogeny (Additional file 5). We did not identify any site under positive selection using SLAC method with a *p*-value threshold of 0.1 (Additional file 5) whereas 139 sites among 178 were under negative selection. FUBAR found evidence of positive selection on one site (#12, posterior probability = 0.992, Additional file 5) and negative selection on 164 sites among 178.

### GH7 phylogeny

**Fig. 6:**
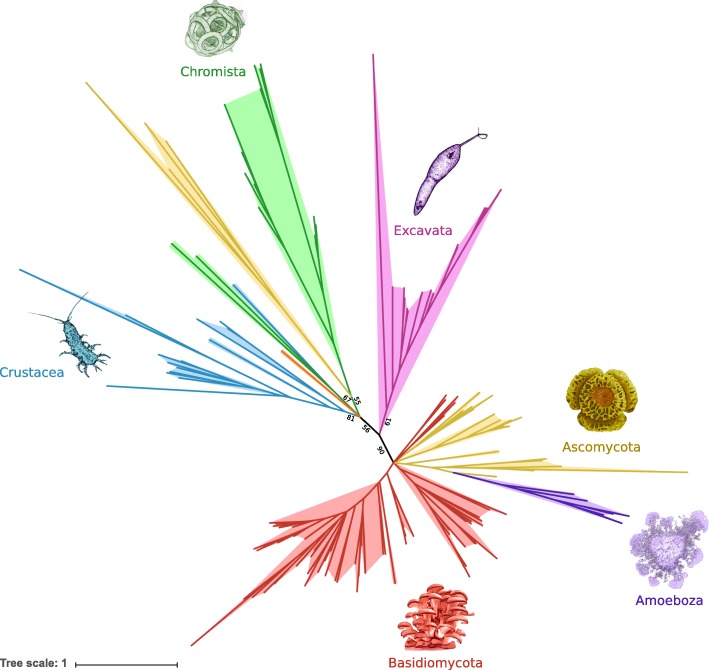
Phylogenetic analysis of the GH7 family from isopods. An unrooted tree shows the relationships between the GH7 proteins of Crustacea (in blue), Demospongea (in orange), Amoeboza (in purple), Basidiomycota (in red), Excavata (in pink), Chromista (in green) and Ascomycota (in yellow). All branches are drawn to scale as indicated by the scale bar. Fast bootstrap values (*n* = 1000) for the main branches are shown as percentages

Protein BLAST searches against the non-redundant protein database of NCBI using GH7 proteins identified in isopod transcriptomes showed a large number of matches with GH7 sequences isolated from fungi (basidiomycetes and ascomycetes), and apart from crustaceans, there was no match with GH7 from any other animals. The comparison of isopod GH7 protein sequences with their homologues in other crustaceans, fungi, oomycetes, protists, amoebozoa and demosponges, showed that they were nested in a clade comprising all the proteins of crustaceans and oomycetes (Chromista), and some proteins of ascomycetes (Fig. 6). This clade was separated from the other clades formed by the GH7 from protists, ascomycetes and basidiomycetes.

## Discussion

The acquisition of lignocellulose degrading CAZymes in isopods could have helped them to better use their diet and favoured their adaptation to the environment, especially during land colonization. The 64 isopod transcriptomes analysed in this study enabled the identification of 205 CAZy families in total and 40 lignocellulose degrading CAZymes, including endogenous cellulases, hemicellulases and LMEs. The resulting CAZomes identified from various freshwater, marine and terrestrial isopod species, highlight different repertoires. Isopods have a great number of CAZymes, placing them at the same level as notorious decomposers such as termite or fungi in terms of CAZymes diversity [41, 42].

Previous studies have shown that the higher abundance of CAZymes in saprophytic fungi is linked to their plant-based nutrition, while lignocellulose degrading CAZymes are less abundant in endophytic fungi or fungi parasitizing animals [43, 44]. We showed that isopods have also been subjected to CAZyme gains and losses, especially as concerns lignocellulose degrading CAZymes. The number of gain events exceeded the number of loss events in terrestrial isopods, indicating that lignocellulose degrading CAZymes acquisition is dominant in this group. On the contrary, lignocellulose degrading CAZymes loss is dominant in aquatic isopods (suborders Asellota, Limnoriidea, Sphaeromatidea and Valvifera). Our results as well as previous studies strongly suggest that some endogenous cell-wall degrading enzymes have been acquired for a long time [40, 45, 46], explaining why some marine isopods could efficiently degrade cellulose and hemicellulose, and in most cases without the contribution of microbiota [26, 27]. Fossil records and phylogenomic analysis dated the origin of terrestrial isopods (suborder Oniscidea) at 300 Mya, which coincides with the diversification of vascular plants on land [47]. To deal with terrestrial plant cell walls that are relatively indigestible [48], a large enzymatic repertoire is necessary. Such conditions would have likely favored the expansion of endogenous CAZomes and the acquisition of digestion-enhancing microbiota in terrestrial isopods to cope with food resources diversification [16, 25, 49]. Gene acquisition seems to be an ancient but still ongoing process in the terrestrial isopods, given the high number of events we observed and the fact that most of them were distributed across the subclade grouping all Oniscidea in the phylogenetic tree. This suggests that a more complete lignocellulose degrading CAZymes repertoire was selected in the terrestrial species. In comparison, freshwater isopods have a less expanded CAZomes. However they could compensate by feeding on detritus that have been partially digested by microbial enzymes [20].

Cellulose and hemicellulose being common resources for most isopods, it is not surprising that both cellulases and hemicellulases were identified in all the transcriptomes. Only three hemicellulases (two xyloglucanases and one mannase) were found in almost all transcriptomes. However, the degradation of hemicellulose, which has a variable composition both within and between plant tissues and species, requires a large enzymatic arsenal. Hence, the other enzymes necessary for this process in isopods could be brought by the microbiota, as shown in *A. vulgare* in which the microbiota plays the major role in hemicellulose degradation [25]. The cellulases belonging to endo-β-1,4-glucanases (EC 3.2.1.4), found to be widespread in isopods, are classified in the GH9 family, an ancient and common eukaryotic gene family [45, 46]. We showed in this study that GH9 sequences are highly conserved in isopods. They were thus duplicated many times in a conservative manner in isopods allowing them to increase the number of expressed enzymes and thus to increase the rate of cellulose digestion. Several other crustacean species are known to express many GH9 genes, like the crayfish *Cherax quadricarinatus* [50] or the land crab *Gecarcoidea natalis* [51] which possess twenty and three forms of GH9 respectively. Furthermore, we identified β-glucosidases (EC 3.2.1.21) affiliated to GH5 family in 49 out 64 isopod transcriptomes, and lytic polysaccharide monooxygenases (AA15) in 61 transcriptomes. Lytic polysaccharide monooxygenases belong to a CAZy family known for its role in chitin remodeling, and they were recently demonstrated to be involved in cellulose oxidation in some arthropods [40]. This is the first record of lytic polysaccharide monooxygenases in isopods, and because several AA15 modules were identified in isopod transcriptomes, we might suppose that they were expanded in isopods to play a role in cellulose degradation like in some other arthropods. Thus, after a mechanical fragmentation of the food that facilitates enzyme access to lignocellulose [25], the synergistic action of high numbers of endo-β-1,4-glucanases, β-glucosidases and lytic polysaccharide monooxygenases would enable isopods to degrade and ingest the cellulose.

In addition to endoglucanases and glucosidases, exoglucanases, belonging to the GH7 family, were found in four isopod species: *Limnoria tripunctata, Proasellus cavaticus*, *Proasellus karamani*, and *Sphaeroma terebrans*. Exoglucanases are required by fungi to degrade the cellulose [52], but they are uncommon in other eukaryotes and prokaryotes. It is known that a small number of crustaceans [22, 26, 27, 53, 54] possesses exoglucanases, constituting rare examples in animals [24]. In our phylogenetic analysis, all the GH7s from crustaceans clustered with oomycete’s GH7s, suggesting an ancient acquisition of exoglucanases in crustaceans, perhaps through horizontal gene transfers with oomycetes as putative donors [26]. Indeed, such transfers of lignocellulose degrading enzymes have already been shown in nematodes [55–57] and insects [58–61]. Alternatively, if the GH7 family was present in the common ancestor of all crustaceans, it was then lost in most isopods.

Lignin being restricted to terrestrial plants, terrestrial isopods are expected to have the capacity to break down this cross-linked phenolic polymer [16]. Its oxidation needs LMEs that are classified in AA families. These enzymes are widespread in fungi and bacteria [62], but very few animals are known to express LMEs. Terrestrial isopods are able to undertake an oxidative degradation of the phenolic compounds [63, 64], and the expression of endogenous laccases (EC 1.10.3.2) and cellobiose dehydrogenases (CDH; EC 1.1.99.18) has been demonstrated in the hindgut of the pill bug *A. vulgare* [25]. Our results showed that CDH are widespread in all Oniscidea and rapidly expanding in this group, while laccases are restricted to some species. Cellobiose dehydrogenases belonging to the AA3 family, are lignin-degrading auxiliary enzymes that assist the activity of the other AAs or support the action of the GHs [62, 65]. They are unable to degrade lignin on their own, but they are known to be involved in the breakdown of cellulose in some fungi. On the other hand, CDH are not widespread in arthropods. They have not been identified in insects, where CAZymes belonging to AA3 family are actually implicated in growth and immunity [66, 67]. In fungi, the multiplicity of enzymatic functions of the AA3 family is reflected by the multigenicity of the AA3 genes [61]. Our study suggests that evolution of the AA3 genes is under episodic diversifying selection in isopods. Moreover, we showed the expansion of the AA3 family genes in Oniscidea: thus, gene duplication events followed by gene diversification might lead to the acquisition of CDH. Their role remains unclear, but they are very likely to participate to the lignocellulose degradation in terrestrial isopods.

In contrast, we showed that AAs are rarer in aquatic isopods and no LMEs were identified in most of the sampled transcriptomes. However, members of the Asellota should also have the capacity to oxidize lignin because they exploit the same food sources as most Oniscidea. The LMEs in Asellota could therefore be of microbial origin, as suggested by Zimmer and Bartholmé [20] upon showing that the activity of hepatopancreatic phenol oxidases is reduced after treatment with antibiotics. Most marine isopods feed on seaweeds, essentially composed of cellulose and hemicellulose but also of phenols (e.g. brown algae) for some of them [49, 68]. However the ability to oxidize phenols is not widespread in marine isopods; Zimmer et al. [17] showed that *Gnorimosphaeroma oregonense* (Sphaeromatidea) has the ability to oxidize phenols while *Idotea wosnesenskii* (Valvifera) has not, despite a diet rich in phenols. *Sphaeroma terebrans* is the only marine species where we predicted an endogenous CDH. While our data suggest that it could degrade the lignocellulose from the wood, Si et al. [69] showed that wood is an unlikely food source for this marine wood-boring isopod and serve only as a source of shelter. Its nutrition could derive from a microphagous filter-feeding habit. Interestingly, it was shown that marine wood-feeding isopods of the genus *Limnoria* (Limnoriidea) can degrade lignin with the help of its hemocyanins that are activated into phenoloxidases [26, 27]. The presence of hemocyanin genes was investigated using sequence similarity searching, and unsurprisingly all transcriptomes show high numbers of hemocyanins. Nevertheless, our data do not allow us to conclude about their potential role in lignocellulose degradation. Hence, marine isopods seem to have a number of strategies to degrade phenols. The ability to degrade lignin could be an important pre-adaptation to terrestrial life [17, 49], which would explain why the ability to degrade phenols alone may have evolved independently in marine isopods.

## Conclusion

Several strategies for lignocellulose degradation have evolved within isopod species. Marine, freshwater and terrestrial isopods showed different CAZomes, the latter having probably significantly evolved during the lifestyle transition from water to land. Similarly, isopod ecology and evolution could have driven the distribution of their endogenous CAZymes, and the microbiota associated to lignocellulose degradation could also influence and be influenced by host CAZomes. For example, a functional complementarity between the host and its microbiome for lignocellulose degradation has been highlighted in the terrestrial isopod *A. vulgare* [25]. Taking the hologenome concept into consideration (i.e. the sum of the host genome and its microbiome), variations in the hologenome can be due to changes in either the host genome or the microbiome [70]. From our results, it appeared clearly that terrestrial and freshwater isopods cannot digest lignocellulose by themselves. To respond to environmental changes, the microbiome can adjust more rapidly than its host, bringing a higher contribution to collective functions in the hologenome. It is therefore important to consider both the microbiome and the evolution of host CAZomes to understand the successful colonization of land by isopods.

## Methods

### Biological samples

Transcriptomic data of 48 isopod species were retrieved from SRA archive (Additional file 1). For each of them, we ascertained that all tissues were represented or that data were generated from whole individuals. Additionally, we generated transcriptomes from 27 isopod species sampled from our laboratory rearing or collected from various field sites in 2015 and 2016 (Additional file 1). By combining these newly generated data to already publicly available data, we finally obtained transcriptomes from 64 different isopod species including new transcriptomic data for 16 species.

### RNA extraction and sequencing

To extract total RNA from the 27 collected isopod species, whole individuals were frozen in liquid nitrogen and ground with a mortar and pestle. Total RNA was then extracted using the RNeasy kit (Qiagen) and treated with RNase-free DNase I (Qiagen), according to the manufacturer’s protocols. After quantification with NanoDrop™ technology, the RNAs were stored at − 80 °C.

The 125 bp paired end sequencing of the extracted RNAs from 27 isopod species were performed on a HiSeq 2500 using Illumina technology (Additional file 1). Each library was constructed with the total RNA of a pool of five males and five females (except for *Trichoniscus pusillus* for which only one male could be sampled). The poly-A selection of the mRNA and the sequencing were carried out by Eurofins (https://www.eurofinsgenomics.eu/).

### Transcriptome assembly

Read quality was checked with FastQC (version 0.11.2; http://www.bioinformatics.babraham.ac.uk/projects/fastqc). Removal of sequencing adaptors and low quality bases was performed with Trimmomatic (version 0.32; [71]). Reads shorter than 35 bp were discarded. Pre-processed reads from each species were assembled using IDBA-Tran [72] with default parameters. Transcript redundancy was removed by clustering with ≥95% identity using CD-HIT-EST (version 4.6; [73]). The completeness of the resulting assemblies was assessed with BUSCO (version 3.0.1; [74]) referring to core arthropod genes.

### Carbohydrate-active enZyme annotation

CAZymes were identified using the Carbohydrate Active enZymes (CAZy) database [24]. Prior to identification, all open reading frames (ORFs) were predicted from transcriptomes using Transdecoder (version 3.0.1; https://transdecoder.github.io/) with default parameters. Subsequently, dbCAN [75], a database which uses hidden Markov Models to define the signature domains for each CAZy family (i.e. Glycoside Hydrolases (GHs), Glycosyl Transferases (GTs), Polysaccharide Lyases (PLs), Carbohydrate Esterases (CEs), Auxiliary Activities (AAs) and Carbohydrate-Binding Modules (CBMs)), was used to identify CAZymes. All predicted ORFs were analysed with dbCAN (October 1, 2017) using HMMER (version 3.1b2; [76]) with an E-value threshold of 0.0001.

To remove CAZymes originating from host microbiota including fungi, prokaryotes and viruses, ORFs identified as CAZymes were compared with the Non-Redundant Protein database (October 1, 2017) using BLASTP [77] with an E-value cut-off of 0.0001. The BLAST outputs were then imported into MEGAN6 software (version 6.9; [78]) for taxonomic assignment using the NCBI taxonomy database. All ORFs assigned to fungi, prokaryotes or viruses were discarded. To verify that all non-host CAZymes were discarded, the 37,481 remaining ORFs were compared again with the latest Non-Redundant Protein database (Avril 25, 2019), using DIAMOND [79] with the “--more-sensitive” mode and an E-value cut-off of 0.001. Among them, 35,194 were assigned to metazoans by MEGAN6 software (version 6.9; [78]), 120 to eukaryotes, 488 to cellular organisms, and 8 were not taxonomically assigned. The 1671 remaining ORFs, most of them being CBMs (1515 including 1068 CBM14), have no hits in the Non-Redundant Protein database. We are aware that this method could exclude sequences that may have been the result of recent horizontal gene transfer. However, without any further genomic information we cannot identify such transfers. The resulting host CAZymes were then imported into Hotpep [80] to predict their enzymatic activity.

CAZyme counts (note that “CAZyme” refers to functional modules or domains, not genes) were normalized to compare their diversity across isopod species. This was done to even out the heterogeneity arising from differential transcriptomic sampling and sequencing methods. CAZyme counts for each transcriptome were divided by the number of ORFs in the transcriptome of interest to calculate the relative abundance for each CAZyme family. Then, normalized count of each family in each transcriptome was calculated by multiplying the relative abundance by 15,256, representing the lowest number of ORFs identified in our dataset corresponding to the *Proasellus ebrensis* transcriptome*.* In summary, the normalization was calculated as follows:

$$ \frac{\begin{array}{c}\mathrm{number}\ \mathrm{of}\ \mathrm{CAZymes}\ \mathrm{in}\ \mathrm{a}\ \mathrm{transcriptome}\ \mathrm{of}\ \mathrm{in}\mathrm{terest}\times \mathrm{number}\ \mathrm{of}\ \mathrm{ORFs}\ \mathrm{in}\ \mathrm{the}\ \mathrm{transcriptome}\ \mathrm{of}\ \\ {}P. ebrensis\ \end{array}}{\mathrm{number}\ \mathrm{of}\ \mathrm{ORFs}\ \mathrm{in}\ \mathrm{a}\ \mathrm{transcriptome}\ \mathrm{of}\ \mathrm{in}\mathrm{terest}} $$

Normalized counts of CAZymes were then used for hierarchical clustering using R (version 3.4.0; http://www.R-project.org/) and Principal Component Analysis (PCA) using ClustVis [81].

The species tree was inferred with Orthofinder2 (version 2.2.7; [82]) according to the STAG (Species Tree Inference from All Genes) method that infers a species tree from sets of multi-copy gene tree [83]. Next, the variation (expansions and contractions) of the CAZyme gene family sizes were analysed using CAFE (version 4.2; [84]). CAFE estimates the global birth and death rate of CAZy families considering their sizes in the extant species and then infers the most likely gene family size at all internal nodes of the phylogeny. Only CAZy families that were shared by more than two species were kept for this analysis.

### GH9 and AA3 phylogenies

Selected sequences which were present in most sampled isopods were examined in depth to infer their phylogeny. Enzymes belonging to GH9 and AA3 families were identified in 97 and 89% of the transcriptomes. All GH9 sequences used for the phylogeny were predicted by Hotpep to function as endo-β-1,4-glucanases (EC 3.2.1.4). Not all the activities of AA3 sequences were predicted and only 54 sequences were predicted to be cellobiose dehydrogenases (EC 1.1.99.18). Consequently, the AA3 sequences were compared to the Pfam database (version 32.0; [85]) using hmm-search (version 3.1b2; [76]) with an E-value cut-off of 0.00001 to identify conserved domains. In a conservative approach, all sequences lacking the conserved domain GMC oxidoreductase (an enzyme family that includes cellobiose dehydrogenases) were discarded. All remaining gene sequences (251 AA3 and 128 GH9) were aligned with Muscle [86] and the subsets of sites used for the phylogenetic analyses were determined with Gblocks using Less Stringent Selection parameters [87]. Phylogenies were constructed with the iqTree software (version 1.6.2; [88]) using ModelFinder for model selection [89] and performing 1000 ultrafast bootstrap with UFBoot [90]. For the construction of the phylogenetic trees, ModelFinder selected the GTR + F + R7 and the TIM2 + F + R6 substitution models for AA3 and GH9, respectively. Trees were modified with the iTol online tool [91]; branches were collapsed when the ultrafast bootstrap values were less than 50. When very close sequences of a given isopod species branched together in the tree, only one sequence was kept. The GH9 tree was rooted with an endo-β-1,4-glucanase from the termite *Coptotermes acinaciformis* (GenBank accession number: AAK12339.1) and the AA3 tree was rooted with a cellobiose dehydrogenase from the fungus *Neurospora crassa* (GenBank accession number: EAA28998.1).

### Assessing positive selection

Multiple tests for selection were performed using the Datamonkey 2.0 web application [92] through i) gene-wide, ii) polymorphic-based and iii) codon-based approaches. The BUSTED (Branch-site Unrestricted Statistical Test for Episodic Diversification) test [93] was used to identify which branches of the phylogeny were under positive selection. We also performed a set of codon-based tests which aim to identify sites under selection pressure by estimating the rates of nonsynonymous (dN) and synonymous (dS) changes at each site in the sequence alignment: i) SLAC (Single-Likelihood Ancestor Counting method, [94]) that uses a combination of maximum-likelihood (ML) and counting approaches and ii) FUBAR (Fast Unconstrained Bayesian Approximation for inferring selection; [95]) that uses a Bayesian approach, assuming that the selection pressure for each site is constant along the entire phylogeny.

### GH7 phylogeny

Unlike GH9 and AA3, GH7 enzymes were identified in only four transcriptomes (*L. tripunctata, P. cavaticus*, *P. karamani*, and *S. terebrans*). To infer their putative origin, a phylogenetic tree was constructed with GH7 sequences from other organisms. A BLASTP using GH7 proteins identified in isopod transcriptomes as query was applied to identify GH7 homologues in the NR protein database of NCBI. The GH7 protein sequences were also retrieved from GenBank with GH7 Pfam id (pfam00840 and cl21662) as query and from the CAZy database (http://www.cazy.org/). Methods used for alignment and phylogenetic tree reconstruction were the same as described above. The WAG+F + R6 substitution model was selected for phylogenetic tree constructions. Branches were collapsed when the ultrafast bootstrap values were less than 50.

## Additional files

Additional file 1: Metrics of samples and associated transcriptomes. (XLSX 19 kb)

Additional file 2: Assembly completenesses assessed with BUSCO referring to core arthropod genes. (PNG 411 kb)

Additional file 3: Protein sequences of CAZy genes identified in the transcriptomes. (FA 16407 kb)

Additional file 4: List of CAZymes identified in the transcriptomes. (XLSX 64 kb)

Additional file 5: Gene-wide and individual site tests for positive selection assessed with BUSTED, SLAC and FUBAR. (XLSX 323 kb)

## Data Availability

Reads used for transcriptomes assemblies are available from the NCBI Sequence Read Archive under accession numbers provided in the Additional file 1. Identified CAZymes are provided in FASTA format in the Additional file 2.

## Abbreviations

AA: Auxiliary Activities
CAZyme: Carbohydrate-Active enzyme
CBM: Carbohydrate Binding Module
CDH: Cellobiose DeHydrogenase
CE: Carbohydrate Esterase
EC: Enzyme Commission number
GH: Glycoside Hydrolase
GT: Glycosyl Transferase
LME: Lignin modifying enzyme
ML: Maximum likelihood
NCBI: National Center for Biotechnology Information
ORF: Open reading frame
PCA: Principal Components Analysis
PL: Polysaccharide Lyase
RNA: RiboNucleic Acid
SRA: Sequence Read Archive
