# Supplementary figures and images for: Lignocellulose degradation in isopods: new insights into the adaptation to terrestrial life
Source: BMC Genomics. 2019 Jun 7;20:462. doi: 10.1186/s12864-019-5825-8 (PMC6555040; doi:10.1186/s12864-019-5825-8)

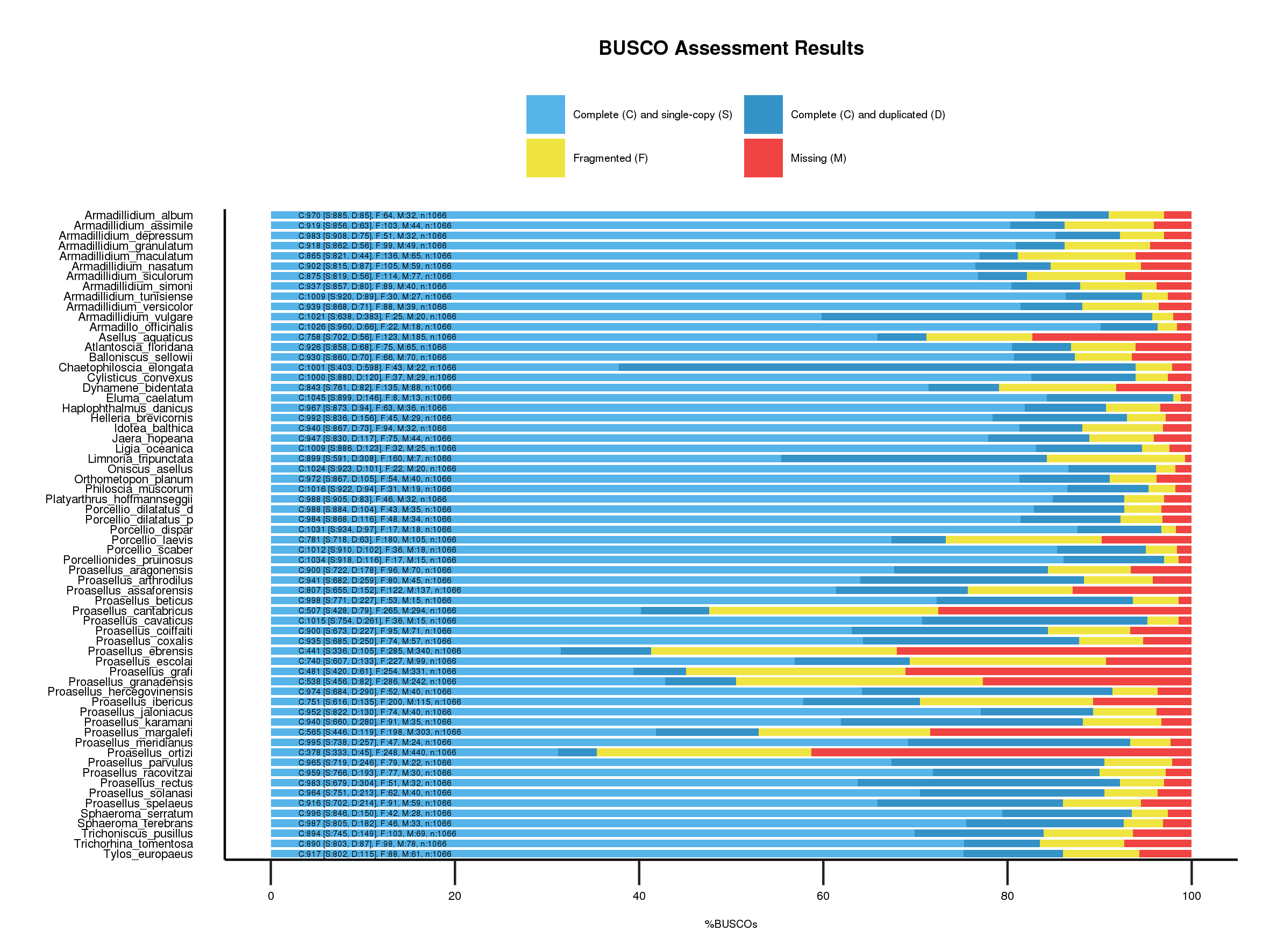

Supplement: Supplementary file 2 — Assembly completenesses assessed with BUSCO referring to core arthropod genes. (PNG 411 kb) [file 12864_2019_5825_MOESM2_ESM.png]
